# Domain Dissection of AvrRxo1 for Suppressor, Avirulence and Cytotoxicity Functions

**DOI:** 10.1371/journal.pone.0113875

**Published:** 2014-12-01

**Authors:** Haifeng Liu, Qingle Chang, Wenjie Feng, Baogang Zhang, Tao Wu, Ning Li, Fangyin Yao, Xinhua Ding, Zhaohui Chu

**Affiliations:** 1 State Key Laboratory of Crop Biology, Shandong Provincial Key Laboratory of Agricultural Microbiology, Shandong Agricultural University, Tai an, Shandong, PR China; 2 Biotechnology Research Center, Shandong Academy of Agricultural Science, Jinan, Shandong, PR China; Institute of Botany, Chinese Academy of Sciences, China

## Abstract

AvrRxo1, a type III effector from *Xanthomonas oryzae* pv. *oryzicola* (Xoc) which causes bacterial leaf streak (BLS) in rice, can be recognised by non-host resistance protein Rxo1. It triggers a hypersensitive response (HR) in maize. Little is known regarding the virulence function of AvrRxo1. In this study, we determined that AvrRxo1 is able to suppress the HR caused by the non-host resistance recognition of *Xanthomonas oryzae* pv. *oryzae* (Xoo) by *Nicotiana benthamiana*. It is toxic, inducing cell death from transient expression in *N. benthamiana*, as well as in yeast. Among the four AvrRxo1 alleles from different Xoc strains, we concluded that the toxicity is abolished by a single amino acid substitution at residue 344 in two AvrRxo1 alleles. A series of truncations from the carboxyl terminus (C-terminus) indicate that the complete C-terminus of AvrRxo1 plays an essential role as a suppressor or cytotoxic protein. The C-terminus was also required for the avirulence function, but the last two residues were not necessary. The first 52 amino acids of N-terminus are unessential for toxicity. Point mutagenesis experiments indicate that the ATP/GTP binding site motif A is required for all three functions of AvrRxo1, and NLS is required for both the avirulence and the suppression of non-host resistance. The putative thiol protease site is only required for the cytotoxicity function. These results determine that AvrRxo1 plays a role in the complex interaction with host proteins after delivery into plant cells.

## Introduction

Plants are different from animals, lacking mobile defender cells and a somatic adaptive immune system. Instead, plants rely on pathogen-associated molecular pattern (PAMP)-triggered immunity (PTI) which is mediated by pattern recognition receptors (PRRs) to recognise pathogen infection and activate immunity responses [1]. Gram-negative pathogenic bacteria are dependent on type III secretion system (TTSS) to deliver effectors directly into host cells to promote pathogen growth and disease development, which results in effector-triggered susceptibility [2]. When an effector is recognized by the disease resistance (R) protein resulting in effector-triggered immunity (ETI), the effector is defined to be an avirulence (avr) protein [3]. ETI, faster and stronger than PTI, usually elicits hypersensitive cell death response in the infection site to inhibit the expansion of disease [4]. To avoid ETI, pathogens either shed or diversify the recognized effector genes, or acquire additional effectors that are able to suppress ETI [3]. In some cases, in the absence of an *R* gene, the particular *avr* gene acts as a virulence factor. TTSS effectors are essential for the pathogenicity of many pathogenic bacteria in animals and plants [1]. If the TTSS is defected, such as the PXO99^A^ mutant *hrpC*/*hrcU*, the pathogenic bacterium will significantly reduce its pathogenicity and become to a non-pathogenic one [5].

Rice bacterial leaf streak (BLS), which is caused by *Xanthomonas oryzae* pv *oryzicola* (Xoc), results in considerable yield loss, especially in southern and central China [6]. And rice bacterial blight (BB), caused by *Xanthomonas oryzae* pv *oryzae*, is another serious bacterial disease. There is no rice R gene identified to be strain-specific resistant to Xoc. Over to 37 R genes have been identified in the rice germplasm resistant to Xoo [7]. About 40 TTSS effectors have been identified in Xoo, which may play an important role in the pathogenicity of Xoo [6]. Some transcription activator-like (TAL) effectors, such as *pthXo1*, *pthXo6*, *pthXo7* and *avrXa7* are found to be required for susceptibility [8]–[14]. Except for the TAL effectors, nearly 20 non-TAL effectors exist in Xoo, and only a few have been identified involved in virulence [15], [16]. There are two copies of the XopZ gene in the genome of PXO99^A^, and mutations in both copies of XopZ have been demonstrated to reduce virulence in means of lesion length and bacterial multiplication compared with PXO99^A^. In addition, the protein has been demonstrated to suppress the host basal defence by repressing callose deposition in the leaves of *Nicotiana benthamiana* [16]. XopR, located in the plant cell plasma membrane, displays significant reduction virulence once mutated in MAFF311018 and Chinese strain 13751 [17], [18], and it also represses flg22-triggered PTI in *Arabidopsis* [17]. Several other Xoo TTSS effectors, including XopN, XopQ, XopX, and XopZ, have been reported to suppress the innate immunity responses induced by cell wall damage in rice [19]. In addition, XopX from *X. campestris* pv. *vesicatoria* was observed to promote lesion development in non-host plants *Nicotiana benthamiana* [20]. Song et al reported that among the 18 type III effector mutations in Xoo, only XopZ was revealed to reduce virulence in rice, suggesting that there may be redundancy in the function of these non-TAL effectors [20]. It has also been reported that Xoo triggers non-host *N. benthamiana* HR, which is dependent on the induction of the expression of harpin coding gene *hpa1* by *hrpD6* [21].

Until now, very little is known about the virulence function of non-TAL effectors from Xoc in rice. The TTSS effector gene *avrRxo1* was found to elicit a hypersensitive response (HR) and confer non-host defense response to Xoc on maize [22]. AvrRxo1 protein is predicted to contain nine putative myristoylation sites and one putative nuclear localization sequence (NLS). The subcellular localization experiments show that AvrRxo1 is localized to the plasma membrane [22]. In addition, AvrRxo1 was also predicted to contain an eukaryotic thiol (cysteine) protease active site and an ATP/GTP binding site motif A (P-loop) [22]. The cognate R gene, Rxo1,encodes NBS-LRR type protein to recognize AvrRxo1 in maize [23]. It was found that Rxo1 can still recognise AvrRxo1 and elicit HR in rice [23]. Forty Xoc strains collected from different geographical regions were identified to contain avrRxo1 and trigger HR on B73 (*Rxo1/Rxo1*) [22]. This suggests that avrRxo1 is conserved in different Xoc strains. Although Xoo shares more than 91% similarity with Xoc at the DNA level (unpublished data), there is no avrRxo1 gene found in Xoo [22]. In addition, the analogue of avrRxo1 was found in *Xanthomonas campestris pv. vesicatoria* (Xcv) which had been identified to inhibit yeast cell growth and result in cytotoxic to *N. benthamiana* [24]. Recently, the other hypersensitive response and pathogenicity (*hrp*) gene *hrpE3* was identified as a new virulence non-TAL effector which is required for full virulence in Xoc [25].

In this study, a new virulence function was identified with *avrRxo1* that it can suppress the PXO99-triggered HR in non-host *N. benthamiana*. Similarly, we determined that *avrRxo1* from Xoc can also exhibit toxicity to yeast and *N. benthamiana*. Thus, *avrRxo1* has three different functions: avirulence, suppression of non-host HR, and cytotoxicity. Truncation and point mutation experiments were performed to define the domains associated with the three different functions. These results show that the three functions were controlled by different domains.

## Results

### AvrRxo1 of Xoc suppresses non-host HR induction triggered by Xoo

**Figure 1:**
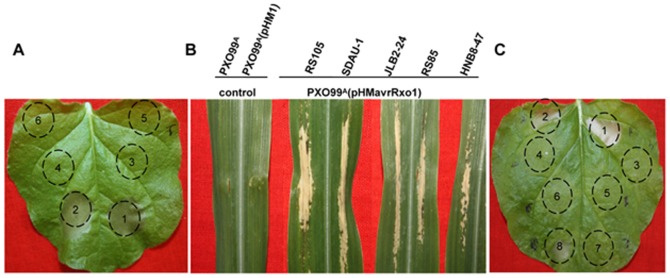
AvrRxo1 was screened out as a suppressor of non-host resistance in *Nicotiana benthamiana*. (A) Symptoms caused by Xoo strains PXO99^A^ (wild type) (1), pHM1 (2), 2C10 (clone of RS105 genomic library) (3), 8E07 (clone of RS105 genomic library) (4), pHMavrRxo1_RS105_ (5) and MgCl_2_ (10 mM) (6) in *Nicotiana benthamiana*. Xoo strains [1×10^8^ colony-forming units (cfu)/mL] were inoculated to *N. benthamiana* (4–6 weeks old) with a needleless syringe, and symptoms were measured at 2 days post-inoculation (dpi). (B) The phenotype of interactions between maize lines B73 and five AvrRxo1 clones: pHMavrRxo1_RS105_, pHMavrRxo1_RS85_, pHMavrRxo1_SDAU-1_, pHMavrRxo1_JSB2-24_ and pHMavrRxo1_HNB8-47_. Xoo strains [1×10^8^ cfu/mL] were infiltrated into B73 (4 weeks old) with a needleless syringe, and symptoms were measured at 2 dpi. Infiltration of B73 with PXO99^A^ containing *avrRxo1* gene results in a hypersensitive response at 2 dpi. PXO99^A^ and PXO99^A^ (pHM1) produced no reaction. (C) Symptoms caused by five AvrRxo1 clones in Xoo strains PXO99^A^ (1) pHM1, (2) pHMavrRxo1_RS105_ (3), pHMavrRxo1_RS85_ (4), pHMavrRxo1_SDAU-1_ (5), pHMavrRxo1_JSB2-24_ (6), pHMavrRxo1_HNB8-47_ (7), and PXO61 (8) in *N. benthamiana*. All experiments were repeated three times with similar results.

As reported, there may exist one or more type III effectors in Xoc that is able to suppress the resistance of plants [26]. In order to clone the suppressor, we hypothesize that it could suppress the HR caused from the recognition of Xoo by *N. benthamiana*. First, we constructed a genomic cosmid DNA library of Xoc strain RS105 and then transformed the library cosmids into the Xoo strain PXO99^A^ one by one. By using an HR reporter system induced by the interaction between PXO99^A^ and *N. benthamiana*, we performed an assay for about 900 PXO99^A^ transformations carrying the library plasmid and *N. benthamiana* (4–6 weeks old). Two clones, carrying cosmid 2C10 and 8E07 respectively, were demonstrated to completely abolish HR on *N. benthamiana* (Fig. 1A), suggesting they carry suppressor genes that inhibit non-host HR. The ends of the inserted DNA fragments of two cosmids were then sequenced. The sequences fell into the same region, and in this region, there is one TTSS protein encoded by the *avrRxo1* gene (data not shown). Interestingly, AvrRxo1 has been reported to be conserved in Xoc. It is able to elicit a non-host HR in maize lines carrying the corresponding *R* gene, *Rxo1* [22]. So we selected *avrRxo1* as the main suppressor candidate gene. The *avrRxo1* gene under its native promoter was then cloned into a pHM1 vector and then introduced into PXO99^A^. The results indicated that PXO99^A^ (pHMavrRxo1) did not trigger HR on *N. benthamiana* (Fig. 1A), suggesting that *avrRxo1* is indeed a suppressor that can suppress the HR mediated by non-host recognition of PXO99^A^ in *N. benthamiana*. To confirm the expression of *avrRxo1* in Xoo, we used the PXO99^A^ (pHMavrRxo1) strain to infiltrate the maize line B73, which carries the *Rxo1* gene. PXO99^A^ (pHMavrRxo1) induced an HR in maize with *Rxo1* (Fig. 1B), suggesting that the *avrRxo1* gene can be expressed normally in *Xoo*.

### The conserved AvrRxo1 in Xoc strains suppresses Xoo-triggered HR in *N. benthamiana*

**Figure 2:**
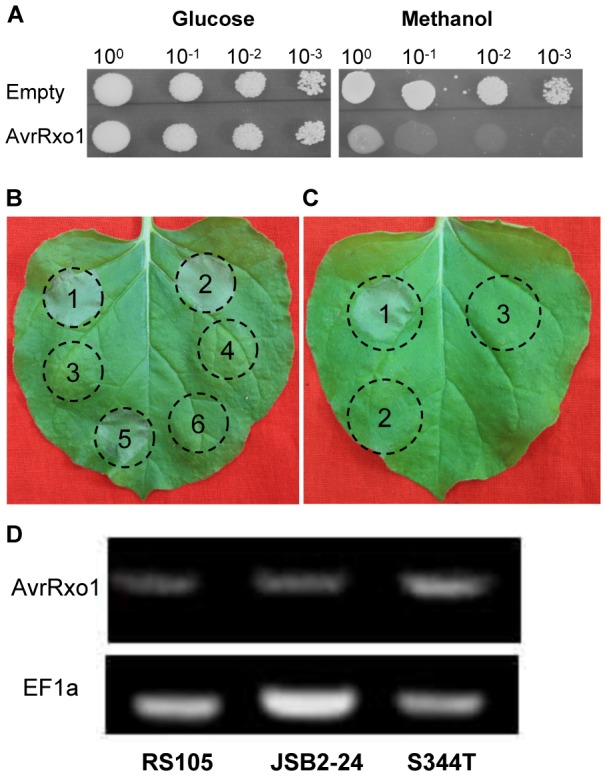
AvrRxo1 induces cell death in *N. benthamiana* and affects cell growth in yeast. (A) Yeast growth is inhibited by expression of AvrRxo1. GS115 yeast strains carrying empty vecter pPIC3.5 or pPIC3.5:avrRxo1_RS105_ were grown overnight in repressing broth (2% glucose) at 30°C. Cultures were then diluted to OD600 = 1.0, and then serial 10-fold dilutions were spotted onto resuppressing or inducing medium (1% methanol). Photographs were taken after 2 days of growth. (B) Phenotype of transient expression of *avrRxo1* from different Xoc strains in *N. benthamiana* (4–6 weeks old). Transient expression of *avrRxo1*_SDAU-1_ (1), *avrRxo1*_RS105_ (2) and *avrRxo1*_RS85_ (5) induces *N. benthamiana* cell death. No cell death for *avrRxo1*_JSB2-24_ (3) and *avrRxo1*_HNB8-47_ (4) as control pGR106 (6) treatment. (C) The serine residue at the 344 position is required for toxicity of avrRxo1 in *N. benthamiana*. The transient expression of avrRxo1 from RS105 (1) induced cell death, whereas avrRxo1 from JSB2–24 (2) did not induce tobacco cell death. AvrRxo1(S344T) (3), whose serine residue at the 344 position was substituted to threonine, failed to induce cell death when transiently expressed in *N. benthamiana*. (D) The normal expression of *avrRxo1* in infected leaves were confirmed by RT-PCR. Housekeeping gene EF1a was selected to normalize the samples. Data shown are representative of three independent experiments with similar results.

In our lab, we collected five Xoc strains from different areas of China, and all of them could induce an HR on B73 (*Rxo1*) (data not shown). Five *avrRxo1* homologs, *avrRxo1*_RS105_, *avrRxo1*_RS85_, *avrRxo1*_SDAU-1_, *avrRxo1*_JSB2-24_, and *avrRxo1*_HNB8-47_, were cloned from the 5 Xoc strains. The AvrRxo1 clones share 98.10% to 100% sequence identity. They also share 98.57% to 100% sequence similarity with AvrRxo1 from BLS256 (Fig. 2). The AvrRxo1 proteins from RS105 and SDAU-1 are identical. Therefore they belong to these four alleles of AvrRxo1. All five PXO99^A^ (pHMavrRxo1) strains could also induce HR on maize B73 (*Rxo1*) (Fig. 1B). These results suggest that all four *avrRxo1* alleles were normally expressed in PXO99^A^, and the interaction with *Rxo1* is conserved. Then, five PXO99^A^ (pHMavrRxo1) strains were infiltrated into *N. benthamiana* separately to test if different AvrRxo1 alleles can suppress the non-host HR in *N. benthamiana*. Compared with the leaves infiltrated with PXO99^A^ (pHM1) that induce HR, there was no HR symptom observed on the leaves infiltrated with all 5 PXO99^A^ (pHMavrRxo1) strains (Fig. 1C). This indicates that the AvrRxo1 allele from different Xoc strains has the ability to suppress the non-host HR triggered by Xoo in *N. benthamiana*. In addition, these results suggest that the function of AvrRxo1 interacts with Rxo1 and suppressing non-host HR is conserved among different Xoc strains.

### AvrRxo1 causes both cell death in *N. benthamiana* and toxicity in yeast

**Figure 3:**
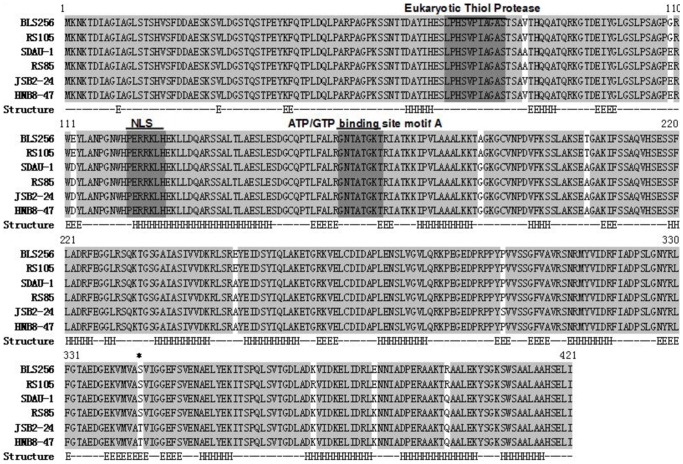
Comparison of amino acids sequences of AvrRxo1 alleles. The grey text indicates identical sequence, and the deep grey indicates putative motifs. The asterisk indicates the residue that is associated with toxicity. The secondary structures under the sequences were predicted using Jpred 3 software. H indicates the α-helix; E indicates β-strand.

Because AvrRxo1 from *X. campestris* pv. *vesicatoria* (Xcv) was found to be toxic and affect cell growth when expressed in yeast [24], a methanol-induced expression assay was adopted to determine if the *avrRxo1* gene from Xoc was also able to produce toxicity when expressed in yeast. Yeast strain GS115 containing pPIC3.5K grew well in the repressing and inducing medium (Fig. 3A). In contrast, GS115 (pPIC3.5K:avrRxo1), with the plasmid carrying the *avrRxo1* gene, grew well in repressing medium but not in inducing medium (Fig. 3A). These results has determined that the AvrRxo1 protein from Xoc is also toxic when expressed in yeast.

Then, a transient expression system was utilised to assess if AvrRxo1 is toxic and able to produce a detectable phenotype in plants. The *avrRxo1* gene from RS105 under the control of the CP (coat protein) promoter was transformed into *Agrobacterium tumefaciens* GV3101 and infiltrated into *N. benthamiana* leaves. Expression of *avrRxo1* resulted in a cell-death symptom 5 days after infiltration in the entire infiltrated area, while *A. tumefaciens* GV3101 containing an empty vector did not induce cell death, even 7 days after infiltration (Fig. 3B). Expression of AvrRxo1 was confirmed by RT-PCR analysis (Fig S1). These findings demonstrate that AvrRxo1 protein is both toxic to yeast and able to induce cell death in *N. benthamiana*.

Then, we tested if the four *avrRxo1* alleles from different Xoc strains are all toxic in *N. benthamiana*. Other four *avrRxo1* clones were inserted into the vector pGR106, and transformed into *A. tumefaciens* GV3101. GV3101 containing empty pGR106 or pGR106:avrRxo1_RS105_ was used as a negative and positive control, respectively. The symptom was observed at day 5 post-infiltration. Similar to *avrRxo1*_RS105_, *avrRxo1*_RS85_, and *avrRxo1*_sdau-1_ induced cell death when transiently expressed in *N. benthamiana*. However, *avrRxo1*_JSB2-24_ and *avrRxo1*_HNB8-47_ did not induce cell death (Fig. 3B). Thus, the toxic function of AvrRxo1 protein is not consistent among different Xoc strains.

### Substitution at residue 344 is involved in toxic function

To explore the abolished toxic function for *AvrRxo1*_JSB2-24_ and *AvrRxo1*_HNB8-47_, further sequence analysis was conducted among four alleles. Compared to AvrRxo1_RS105_, there are 10 and 9 amino acids substitutions in AvrRxo1_JSB2-24_ and AvrRxo1_HNB8-47_, respectively. Except for substitution at the position 344, which is serine in AvrRxo1_RS105_ AvrRxo1_RS85_, and AvrRxo1_sdau-1_ but threonine in AvrRxo1_JSB2-24_ and AvrRxo1_HNB8-47_, other eight common substitutions were also identified in either AvrRxo1_RS85_ or AvrRxo1_sdau-1_ that could induce cell death. This indicated that substitution at residue 344 may be responsive for the abolished toxic function for AvrRxo1_JSB2-24_ and AvrRxo1_HNB8-47_. To confirm the important role of the amino acid at the position 344, the serine of AvrRxo1_RS105_ was substituted with threonine. The AvrRxo1 mutant (S344T) was cloned into the pGR106 vector for toxicity testing in *N. benthamiana*. As shown in Fig. 3C, S344T failed to induce cell death when expressed in *N. benthamiana*, suggesting that the residue 344 is essential for the toxic function of AvrRxo1.

### C-terminus plays an important role in the functions of AvrRxo1

The functions of suppressor and avirulence but not toxicity are conserved in AvrRxo1 alleles from different Xoc strains. It is possible that the three functions may be controlled by different domains. However, the information about function domains is limited in AvrRxo1 protein. The secondary structure of the protein was analysed using the Jpred 3 and GlobPlot software [27], [28]. AvrRxo1 is predicted, with low confidence, to contain an intrinsically disordered N-terminus (Fig. S2), where there are several short extended helical strands and a potential globular domain in the C-terminus that contains six β-strands inserted between and flanked by 10 α-helixs (Fig. S3).

**Figure 4:**
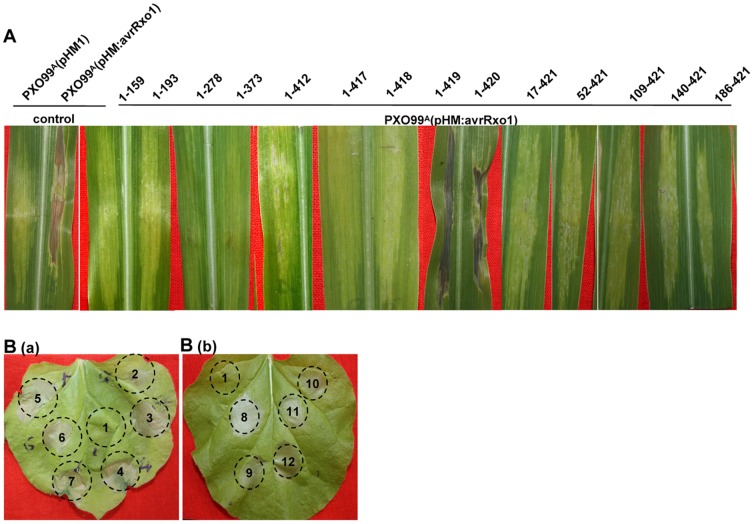
Analysis the contribution of C- and N-terminus to the 3 function of AvrRxo1. (A) The phenotype of interactions between maize lines B73 and deletion mutants of AvrRxo1. Infiltration of B73 with PXO99^A^ containing *avrRxo1* gene results in HR at 2 dpi. PXO99^A^ (pHM1) as negative control. Fragments AvrRxo1(17–421), AvrRxo1(52–421), AvrRxo1(109–421), AvrRxo1(140–421), AvrRxo1(186–421), AvrRxo1(1–159), AvrRxo1(1–193), AvrRxo1(1–278), AvrRxo1(1–373), AvrRxo1(1–412), AvrRxo1(1–417), and AvrRxo1(1–418), abolished induce HR on B73, while fragments AvrRxo1(1–419) and AvrRxo1(1–420) produce HR on B73 at 2 dpi. (B) Suppressions of non-host HR by deletion mutants of AvrRxo1 in *N. benthamiana*. Infiltration of *N. benthamiana* with PXO99^A^ (pHM:avrRxo1) (1) and PXO99^A^ (pHM1) (2) as control. Fragments AvrRxo1(17–421) (3), AvrRxo1(52–421) (4), AvrRxo1(109–421) (5), AvrRxo1(140–421) (6), AvrRxo1(186–421) (7), AvrRxo1(1–412) (8), AvrRxo1(1–417) (9), AvrRxo1(1–418) (10), AvrRxo1(1–419) (11), AvrRxo1(1–420) (12) did not suppress the non-host HR caused by PXO99^A^ in *N. benthamiana*. RT-PCR was used to confirm the expression of *avrRxo1* in inoculated leaves. All experiments were repeated three times with similar results.

**Figure 5:**
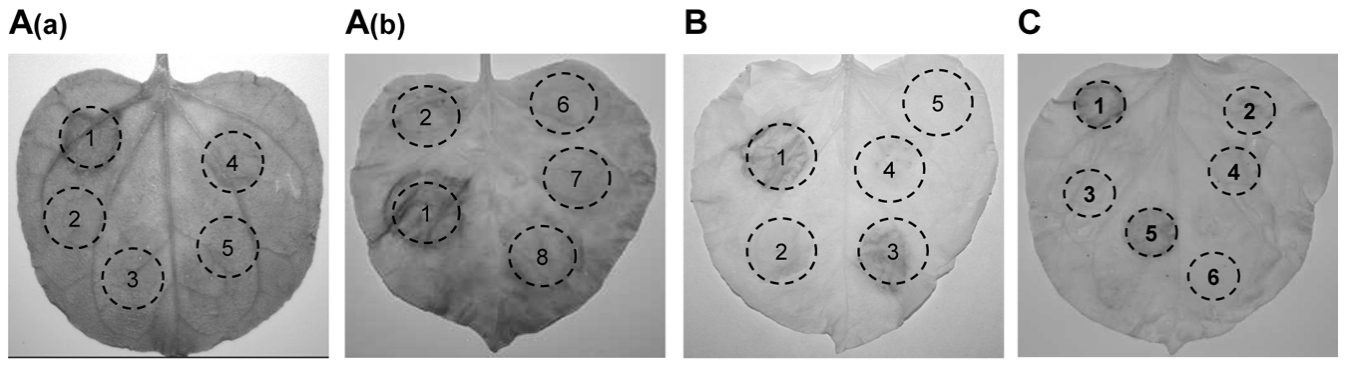
The transient expression of AvrRxo1 mutant proteins in *Nicotiana benthamiana*. The expressions of avrRxo1_RS105_ (1), pGR106 (2) in *N. benthamiana* cells were used as positive and negative controls, respectively. Similarly hereinafter. (A) The C-terminus-truncated proteins AvrRxo1(1–159) (3), AvrRxo1(1–193) (4), AvrRxo1(1–278) (5), AvrRxo1(1–373) (6), AvrRxo1(1–412) (7) and AvrRxo1(1–420) (8) expressed in *N. benthamiana* all failed to induce cell death. (B) The transient expression of N-terminus truncated proteins in *N. benthamiana*. The expressions of AvrRxo1(52–421) (3) can still induce cell death, whereas AvrRxo1(109–421) (4) and AvrRxo1(140–421) (5) loss the toxic ability in *N. benthamiana*. (C) The transient expression of mutant proteins in *N. benthamiana*. Transient expression of avrRxo1(G165A) (3), avrRxo1(K166N) (4), and avrRxo1(H71A) (6) did not induce cell death, however expression of avrRxo1(nls) (5) still resulted in cell death in *N. benthamiana*. The normal expression of *avrRxo1* in infected leaves were confirmed by RT-PCR. All experiments were repeated three times with similar results.

To determine the contribution of the N- and C-terminus to the function of AvrRxo1, we performed a deletion analysis. Site-directed C-terminal deletions at 159, 193, 278, 373, and 412 position were generated and cloned in both pGR106 and pHM1. They they were analysed for their ability to elicit a maize B73 HR, repressing HR triggered by Xoo in *N. benthamiana* and plant cell toxicity. Similar to the negative control, the 5 C-terminal deletions failed to elicit a HR, suppress non-host HR and induce cell death (Fig. 4A). To confirm that the abolished HR was not due to lack of expression of the truncations in Xoo, RT-PCR analysis with specific primer for avrRxo1 were achieved to suggest that all avrRxo1 truncations were successfully expressed at transcription level (data not shown). Based on the aboved results, we performed another four C-terminal deletions resulting in the lack of the last one to four amino acids of AvrRxo1. When the last or the last two amino acids were deleted, AvrRxo1(1–420) and AvrRxo1(1–419) were still recognised by Rxo1, resulting in the induction of HR in maize. But they lost the suppression ability in *N. benthamiana* (Fig. 4A, B). AvrRxo1(1–418), lacking the last three amino acids was unable to be recognised by Rxo1 in maize and suppress the non-host HR in *N. Benthamiana* (Fig. 4A, B). All the C-terminus truncated AvrRxo1 proteins were not toxic to *N. benthamiana* (Fig. 5A). These results suggest that the C-terminus plays an important role in all three functions of AvrRxo1, and a complete C-terminus is required for the suppressor function and toxicity function of AvrRxo1.

A disordered region with an unknown function was predicted in the N-terminus. We also made six N-terminal deletions at the 17, 52, 109, 140, and 186 amino acids and tested the functions of the mutants. All the N-terminal deletions transformed into PXO99^A^ resulted in the loss of recognition by Rxo1 and suppression of non-host HR (Fig. 4A, B). However, when transiently expressed in *N. Benthamiana*, AvrRxo1(17–421) and AvrRxo1(52–421) still induced cell death in *N. benthamiana*, whereas AvrRxo1(109–421), AvrRxo1(140–421), and AvrRxo1(186–421) lost toxicity (Fig. 5B). These results suggest that N-terminus is essential for the function of the AvrRxo1 protein as well as C-terminus.

### The suppressor and avirulence functions are dependent on ATP/GTP binding site motif A but not the cysteine protease active site

**Figure 6:**
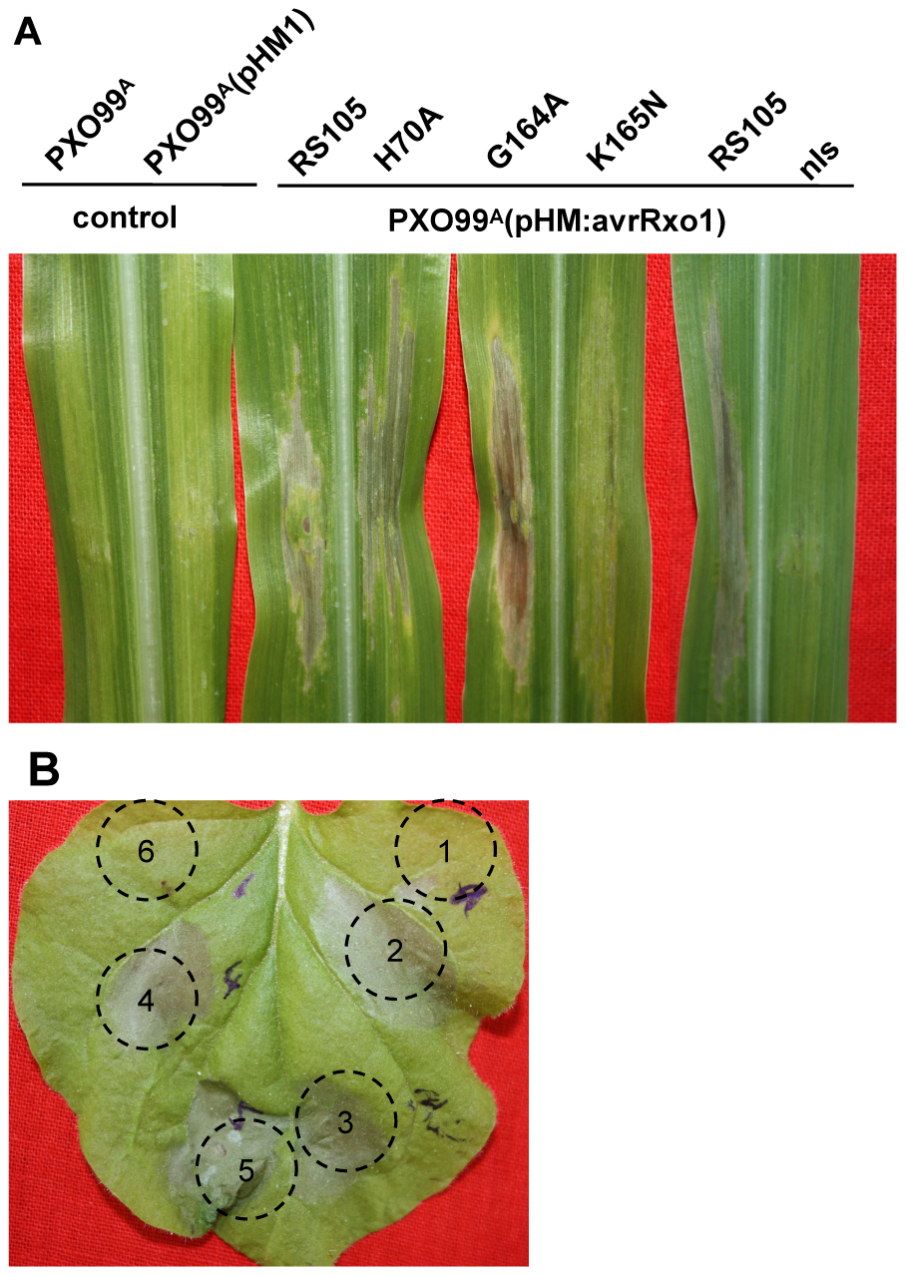
Function analysis of the four AvrRxo1 mutants expresssed in PXO99^A^. (A) The phenotype of interactions between maize lines B73 and four AvrRxo1 mutants: AvrRxo1(H71A), AvrRxo1(G165A), AvrRxo1(K166N), and AvrRxo1(nls). Mutants AvrRxo1(H71A) and AvrRxo1(G165A) induce HR at 2 dpi, however mutants AvrRxo1(K166N) and AvrRxo1(nls) are abolsihed to elicit HR on B73. (B) The phenotype of suppression of non-host HR in *N. benthamiana* caused by PXO99^A^. PXO99^A^(pHM1) was used as negative control and PXO99^A^(pHM:avrRxo1) as positive control. PXO99^A^ containing AvrRxo1(H71A) (6) did not elicit the non-host HR in *N. benthamiana*, while PXO99^A^ containing the fragments AvrRxo1(G165A) (3), AvrRxo1(K166N) (4), and *nls* (5) can still elicit non-host HR. RT-PCR was used to confirm the expression of *avrRxo1* in inoculated leaves. All experiments were repeated three times with similar results.

We further analysed the relationship between conserved motifs and the three functions of AvrRxo1. The putative key sites of the cysteine protease and ATP/GTP binding site motif A were substituted by alanine separately, and the mutants were tested for the three functions of interest. We found that the ATP/GTP binding site motif A mutant (K166N) neither induced HR in maize B73 nor suppressed the non-host HR in *N. benthamiana*, whereas the mutant ATP/GTP binding site motif A mutant (G165A) was still recognised by Rxo1 but lacked suppression ability (Fig. 6A, B). However, the cysteine protease active site mutant (H71A) did not affect the recognition with Rxo1 and suppression of non-host HR (Fig. 6A, B). Thus, the ATP/GTP binding site motif A plays an important role in the recognition with Rxo1 and in acting as a suppressor. The cysteine protease active site may not associated with the two functions of AvrRxo1 proteins. Differently, the cytotoxicity was lost in G165A, K166N and H71A (Fig. 5C). This indicates that the ATP/GTP binding site motif A and the cysteine protease active site were required for the toxicity function of AvrRxo1.

### NLS can import AvrRxo1 into nuclear and is required for suppressor and avirulence functions

**Figure 7:**
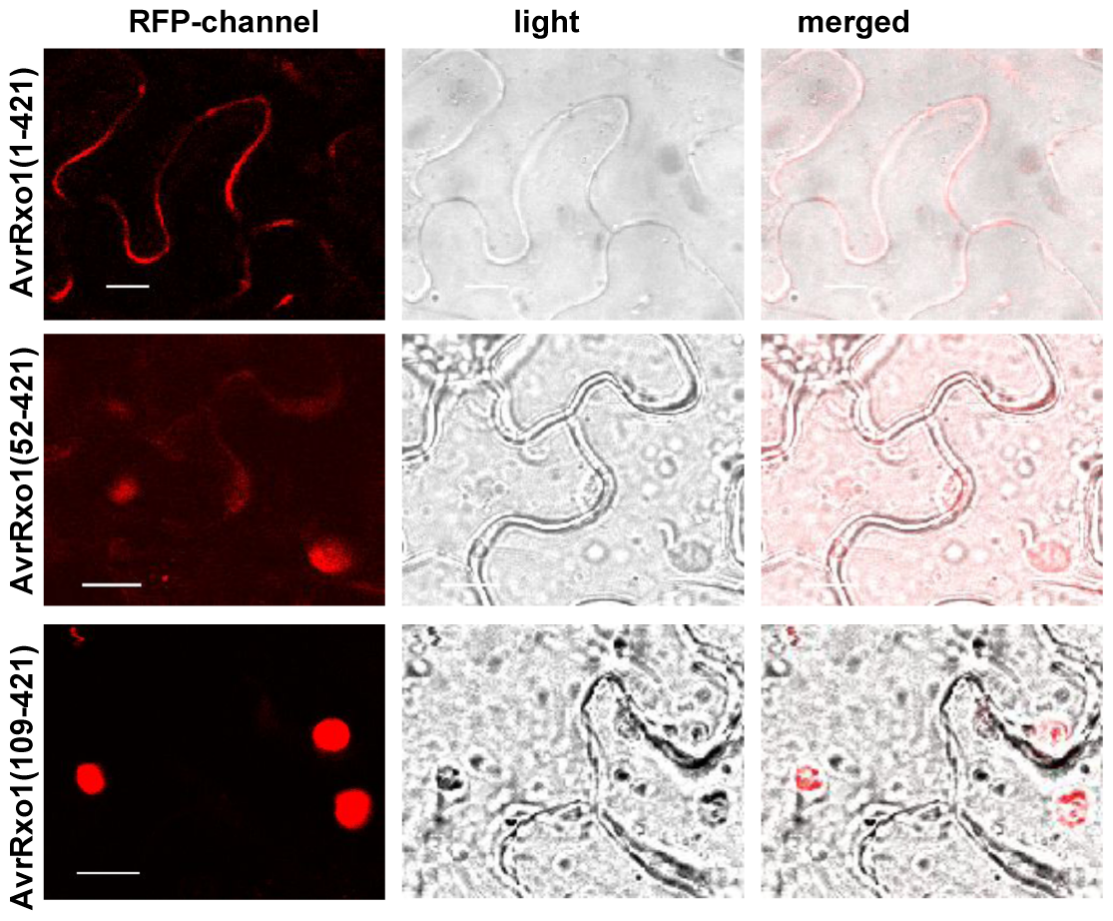
Different fragments of AvrRxo1 in frame with red fluorescent protein (RFP) display different subcellular location. The transient expression of RFP-AvrRxo1(1–421) in *N. benthamiana* revealed that the RFP-AvrRxo1 fusion protein was localised to the plasma membrane. Expressing RFP-AvrRxo1 (52–421) in *N. benthamiana* revealed that the RFP fluorescence was observed both in the plasma membrane and nucleus. The RFP fluorescence of RFP-AvrRxo1(109–421) was observed only in the nucleus. Bars  = 10 µm.

AvrRxo1 was also predicted to contain a putative NLS (122-PERRKLH-128) [22], which may facilitate the transportation of the protein AvrRxo1 into the cell nucleus. To determine the role of NLS, AvrRxo1(1–421), AvrRxo1(52–421) and AvrRxo1(109–421), were fused with red fluorescent protein (RFP) and transiently expressed in 4–6 weeks old *N. benthamiana*. Similar to the results of subcellular of avrRxo1 done in onion cells, the full length avrRox1 is mostly localized to the plasma membrane in *N. benthamiana* cells. The RFP fluorescence of AvrRxo1(52–421)-RFP, which lacks the first two putative myristoylation sites, was observed both in the plasma membrane and nucleus, while AvrRxo1(109–421) lacking the first four myristoylation sites was mostly located in the nucleus (Fig. 7). These results suggest that the NLS has a role in importing AvrRxo1 into the nucleus when the membrane anchor signal is missing.

Given that the NLS has the ability to import AvrRxo1 into the nucleus, its contribution to the three functions was then investigated. We substituted the amino acids RRK essential for the function of NLS by three alanines to obtain a mutant, *nls* (R124A, R125A, K126A). Then it was used to test its ability to be recognised by Rxo1, suppress non-host HR, and toxicity. We found that *nls* (R124A, R125A, K126A) failed to induce HR in B73 and suppress the non-host HR to PXO99 ^A^ in *N. benthamiana* (Fig. 6A, B),but still maintained toxicity when transiently expressed in *N. benthamiana* (Fig. 5C). These results suggest that NLS is an important motif for the function of AvrRxo1, and nuclear localisation is required for resistance mediated by recognition with Rxo1 and suppress the defense response.

## Discussion

Previously, *avrRxo*1 cloned from Xoc was demonstrated to be capable of inducing an HR in the maize line B73, which carries *Rxo1* [22], but little is known about its virulence. As Xoc TTSS effectors have been found to be able to inhibit R gene-mediated defense responses in rice [26], it is unknown whether AvrRxo1 is one of these suppressors. In this study, we identified that two clones containing *avrRxo1* gene can suppress non-host pathogen-induced HR in *N. benthamiana* (Fig. 1). Interestingly, the two clones could also inhibit the host defense response to Xoo controlled by XA21 in rice (data not shown), suggesting it is an effective way to clone suppressors of Xoc in *N. benthamiana*, and *avrRxo1* may be one of the suppressors in Xoc. Meanwhile, the result that the two clones are not able to suppress the resistance of *xa13* suggests that there is another effector in Xoc that can inhibit *R* gene-mediated defense.

**Figure 8:**
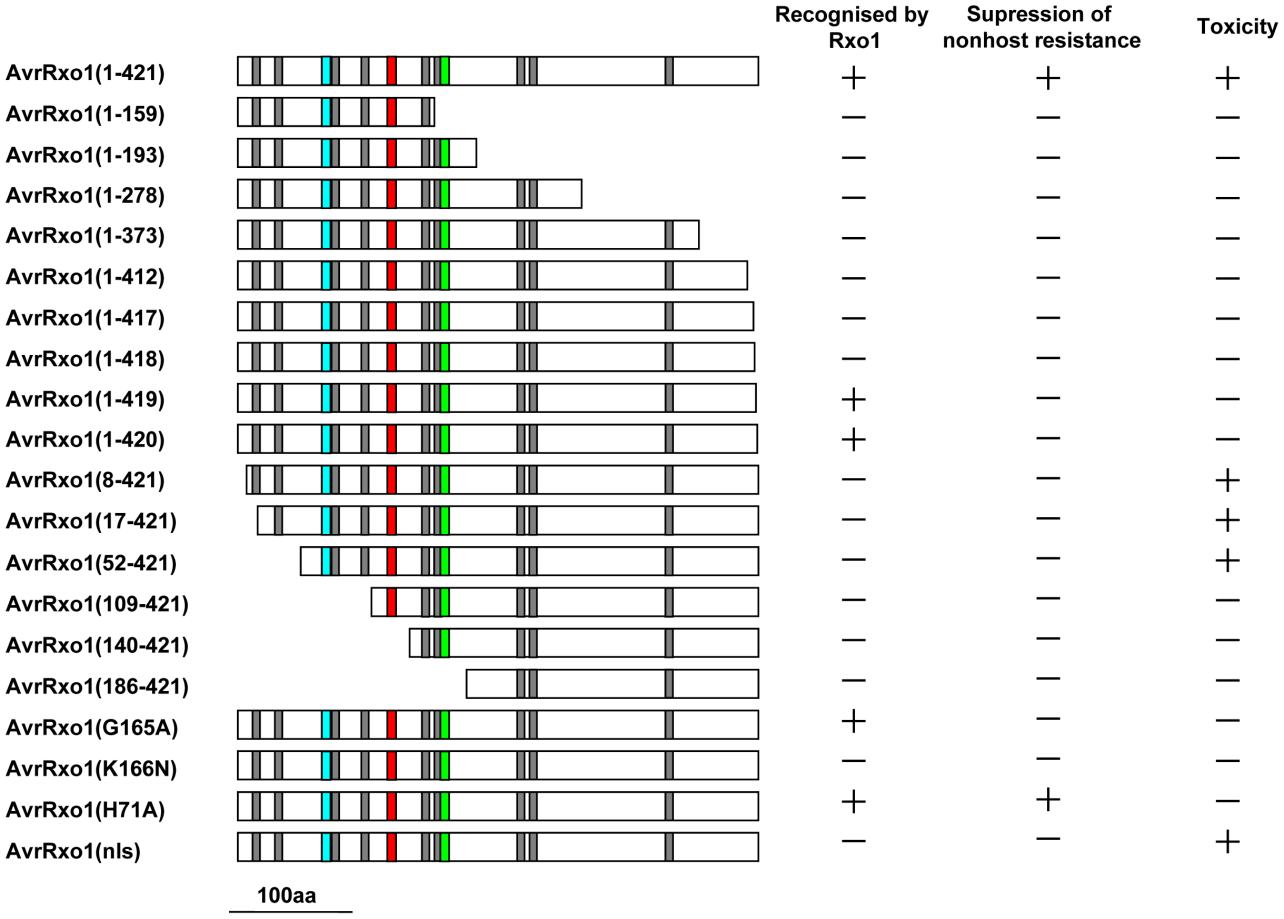
Model of AvrRxo1 mutants function in recognision of Rxo1, suppression of nonhost resistance, and toxin. The colour boxes indicate that: grey, putative myristoylation site; red, nuclear localization sequence; green, ATP/GTP binding sites; blue, cysteine protease motif.

Twenty-six non-TAL TTSS effector genes and 28 TAL effector genes were identified in the Xoc strain BLS256 [29], and most of their functions are unknown. Some non-TAL effectors existing in both Xoo and Xoc were reported to be no virulence function in rice line IR24 [16]. Recently, several Xoo TTSS effectors, XopN, XopQ, XopX, and XopZ, were reported to suppress innate immunity responses in plants [19]. In addition, the TTSS effectors XopD, XopN, XopJ, XopX and others from *X. campestris* pv. *vesicatoria* are also able to suppress the basal defence responses in host or non-host plants [30]–[33]. Thus, future experiments are needed to screen Xoo-resistant rice lines, such as IRBB7, IRBB10 and IRBB13, to identify new suppressors. There are more than 31 TTSS effectors in *Pseudomonas syringe* pv. *tomato* DC3000 (DC3000) [34], and several effectors have been reported to enhance the pathogenicity of bacteria on host plants [35]. Several TTSS effector proteins from DC3000, AvrPto, AvrPtoB, HopPtoD2, and HopPtoN, have been reported to possess HR-suppressing ability in host and non-host plants [36]–[39]. The suppression of cell death by AvrPto is determined by the ability to interact with the R protein Pto [38]. HopPtoN protein, a member of the CA clan of papain-like cysteine proteases, has cysteine protease activity *in vitro* [39]. However, whether the cysteine protease activity is required for the HR-suppressing ability remains to be determined. AvrRxo1 protein has been revealed to contain a eukaryotic thiol (cysteine) protease active site, suggesting that AvrRxo1 may possibly have cysteine protease activity likely the HopPtoN protein that requires future experiments to determine. However, a cysteine protease active site mutant (H71A) that is similar to wild-type AvrRxo1 can still be recognized by Rxo1 to induce HR in maize and inhibit the non-host HR in *N. benthamiana* (Fig. 6, 8). These findings determine that the putative cysteine protease active site is not important for the suppressor function, but it is essential for cytotoxicity function.

BLAST results reveal the homologs of AvrRxo1 in *Xanthomonas campestris* pv. *vesicatoria*, *Xanthomonas axonopodis* pv. *citrumelo* and *Acidovorax citrulli* AAC00-14. Whether these AvrRxo1-like proteins contain similar function in terms of interaction with Rxo1 or suppression of non-host resistance require further determination. It has been reported that *avrRxo1* from Xcv inhibits cell growth by arresting the cell cycle when expressed in yeast [24]. A similar response was also observed in yeast expressing *avrRxo1* and in plants with transient expression of *avrRxo1* from the Xoc strains RS105, SDAU-1, and RS85 (Fig. 3A, 3B). Interestingly, *avrRxo1* from Xoc strains JSB2-24 and HNB8-47 failed to induce cell death when expressed in *N. benthamiana*, although there is highly identical among amino acids sequence (Fig. 3B). Based on the sequence analysis, we speculated that the amino acid at the position 344, that is serine in AvrRxo1_RS105_, AvrRxo1_SDAU-1_, and AvrRxo1_RS85_ but threonine in AvrRxo1_JSB2-24_, and AvrRxo1_HNB8-47_, is essential for toxicity. The BLAST results revealed the same as AvrRxo1_RS105_, AvrRxo1 alleles from Xcv is also serine at the position 379 (equivalent to the position 344 in AvrRxo1 from Xoc). The mutant AvrRxo1(S344T) transient expression in *N. benthamiana* did not induce cell death, confirming our speculation (Fig. 3). Then, we proposed whether the position 344 is a part of an important functional amino acid. The results of secondary structure prediction reveal that the position 344 participates in assembling a β-strand (Fig. 2), except that these findings provide no clues about the important particular role of the position 344.

We determined that the avirulence function of interaction with Rxo1 and suppression of non-host HR is conserved among AvrRxo1 proteins from different Xoc strains. It has been reported that all 40 Xoc tested strains can induce strong HRs in maize with *Rxo1* [22]. These results suggest that AvrRxo1 may play a key role in the virulence of Xoc. G165A and K166N mutants have mutations in the putative ATP/GTP binding site motif A. K166N did not induce HR in maize with *Rxo1* and is disable to suppress non-host HR, while G165A could still interact with Rxo1 but could not suppress non-host HR and lost toxicity (Fig. 5, 6, 8). This suggests that the function of interaction with Rxo1 and suppression of non-host HR is controlled by different amino acid site. In addition, AvrRxo1(1–420), the C-terminal final one amino acid deletion mutant, failed to suppress non-host HR to Xoo in *N. benthamiana* but still recognized by Rxo1 (Fig. 4, 8). This further confirms that the two functions are controlled by different domains.

There is an evidence that some suppressors act upon the signal transduction pathway that controls programmed cell death to suppress the defense-associated HR. The DC3000 effector HopAI1 directly inactivates MPK3 and MPK6 by dephosphorylation and consequently suppresses flg22-induced gene expression, oxidative burst, and callose deposition, resulting in increased disease susceptibility in plants [40]. AvrPto and AvrPtoB directly target FLS2, EFR, and BAK1 to affect receptor kinase complexes to block immune signaling pathway [41], [42]. Using virus-induced gene silencing, 7 ACE (Avr/Cf-elicited) genes were identified to be required for non-host HR to Xoo in *N. benthamiana* [43]. It is believed that oxidative burst and calcium-dependent signaling pathways are important in the non-host resistance to Xoo [43]. Therefore AvrRxo1 is speculated to interfere in the progress of oxidative burst or the calcium-dependent signaling pathways to suppress the non-host HR. Bahadur and Basak reported that a number of residues of AvrRxo1 at the interface of interaction with Rxo1 were in the C-terminal region (379–408) of AvrRxo1 [44]. However, our results show that a complete C terminus is required for the suppressor and cytotoxic function of AvrRxo1. This led us to speculate that the C-terminus may play an important role in interacting with plant target proteins.

The subcellular localisation is important for the function of proteins. The effector protein AvrPto is reported to localise on plant cell membrane [45]. AvrPto mutation of the putative myristoylation site, G2A, abolished the membrane association and resulted in lost suppression activity [45]. The myristoylation motif of myristoylated proteins targeted to the plasma membrane is usually located at the N-terminus of the protein with glycine right after the starting methionine, it is not the case of AvrRxo1 [46]. AvrRxo1 also contains nine putative myristoylation site, however, none of these putative myristoylation site is located at the end of N-terminus. Subcellular location experiments revealed that avrRxo1 is localised on the plasma membrane (Fig. 7) After being truncated at the first two putative myristoylation sites, some AvrRxo1 protein were observed to translocate into nucleus. When losing the four putative myristoylation sites, AvrRxo1 protein is found mainly in the nucleus (Fig. 7), suggesting that the first four myristoylation site lead AvrRxo1 to localise to membrane and the NLS is able to import AvrRxo1 into the cell nucleus. The observations proved that AvrRxo1(8–421), AvrRxo1(17–421) and AvrRxo1(52–421) were shown toxic to *N. benthamiana* cells. These suggest that the membrane localisation is required for toxicity activity. In addition, the *nls* mutant protein is still toxic to *N. benthamiana* cells (Fig. 5C, 8) suggesting that the nucleus localization is not required for the toxic function. AvrBs3 carries two functional NLSs, which are required for HR induction. The NLS2 is required for interaction with CaIMPα1 and CaIMPα2 [47], [48]. The *nls* mutant (R124A, R125A, K126A) lost the ability to elicit an HR in maize carrying the Rxo1 gene, and the mutant lost suppressor activity but it could still induce cell death when transiently expressed in *N. benthamiana* (Fig. 8). These results suggest that nuclear localization is important for the induction of HR in maize and for the inhibition of non-host HR. These results further demonstrate that the three functions of AvrRxo1 are controlled by different domains or amino acid site and can be structurally separated.

## Materials and Methods

### Bacterial strains and plasmids

The bacterial strains and plasmids used in this study are described in Table 1, and the primers used are described in Table S1. The strains of *Escherichia coli* were cultured in Luria-Bertani (LB) at 37°C. *X. oryzae* pv. *oryzicola* and *X. oryzae* pv. *oryzae* strains were grown in peptone-sucrose agar (PSA) at 28°C. *A. tumefaciens* strain GV3101 was cultured in Luria-Bertani (LB) at 28°C.

**Table 1:** Strains and plasmids used in this study.

Strains or plasmids	Relevant characteristics	Reference of source
*Xanthononas oryzae* pv. *oryzae*		
PXO99^A^	Wild-type, Philippine race 6	This laboratory
*Xanthononas oryzae* pv. *oryzicola*		
RS105	Wild-type, Chinese race 2; Rif^r^	Zou *et al*., 2006
SDAU-1	Wild-type, Chinese race isolated from Hubei province; Rif^r^	Feng *et al*., 2013
RS85	Wild-type, Chinese isolate	Feng *et al*., 2013
JSB2-24	Wild-type, Chinese isolate	Feng *et al*., 2013
HNB8-47	Wild-type, Chinese isolate	Feng *et al*., 2013
*Escherichia coli*		
DH5α	F–φ80*lacZ*ΔM15 Δ(*lacZYA-argF)*U169 deoR recA1 endA1 hsdR17 phoA supE44 thi-1 gyrA96 relA1 l thi-1 relA1λ	CWBIO
*Agrobacterium tumefaciens*		
GV3101	Rif^r^	This laboratory
Plasmids		
pHM1	Broad-spectrum cosmid vector that replicates in X. *oryzae* pv. *oryzicola*, *X. oryzae pv. oryzae*, and *E. coli*; Sp^r^, Sm^r^, cos, parA, IncW, derivative of R140	Yang *et al*., 2006
pGR106	Km^r^, binary PVX	Takken *et al*., 2000
pPIC3.5K	5′ *AOX1*; 3′ *AOX1*; *HIS4*; Gm^r^, Amp^r^	Invitrogen
pGEM-T Easy	Amp^r^; *lacZ*	Promega
pHMavrRxo1	AvrRxo1 from different Xoc strains under the native promoter of *avrRxo1*; Sp^r^, Sm^r^	This work
pHMavrRxo1_(1-159)_	The first 159 amino acids of AvrRxo1 from RS105 expressed in pHM1 under the native promoter of avrRxo1; Spr, Smr	This work
pHMavrRxo1_(1-193)_	The first 193 amino acids of AvrRxo1 from RS105 expressed in pHM1 under the native promoter of avrRxo1; Spr, Smr	This work
pHMavrRxo1_(1-278)_	The first 278 amino acids of AvrRxo1 from RS105 expressed in pHM1 under the native promoter of avrRxo1; Sp^r^, Sm^r^	This work
pHMavrRxo1_(1-373)_	The first 373 amino acids of AvrRxo1 from RS105 expressed in pHM1 under the native promoter of avrRxo1; Sp^r^, Sm^r^	This work
pHMavrRxo1_(1-412)_	The first 412 amino acids of AvrRxo1 from RS105 expressed in pHM1 under the native promoter of avrRxo1; Sp^r^, Sm^r^	This work
pHMavrRxo1_(1-417)_	The first 417 amino acids of AvrRxo1 from RS105 expressed in pHM1 under the native promoter of avrRxo1; Sp^r^, Sm^r^	This work
pHMavrRxo1_(1-418)_	The first 418 amino acids of AvrRxo1 from RS105 expressed in pHM1 under the native promoter of avrRxo1; Sp^r^, Sm^r^	This work
pHMavrRxo1_(1-419)_	The first 419 amino acids of AvrRxo1 from RS105 expressed in pHM1 under the native promoter of avrRxo1; Sp^r^, Sm^r^	This work
pHMavrRxo1_(1-420)_	The first 420 amino acids of AvrRxo1 from RS105 expressed in pHM1 under the native promoter of avrRxo1; Sp^r^, Sm^r^	This work
pHMavrRxo1_(17-421)_	The fragment from 17 to 421 amino acids of AvrRxo1 from RS105 expressed in pHM1 under the native promoter of avrRxo1; Sp^r^, Sm^r^	This work
pHMavrRxo1_(52-421)_	The fragment from 52 to 421 amino acids of AvrRxo1 from RS105 expressed in pHM1 under the native promoter of avrRxo1; Sp^r^, Sm^r^	This work
pHMavrRxo1_(109-421)_	The fragment from 109 to 421 amino acids of AvrRxo1 from RS105 expressed in pHM1 under the native promoter of avrRxo1; Sp^r^, Sm^r^	This work
pHMavrRxo1_(140-421)_	The fragment from 140 to 421 amino acids of AvrRxo1 from RS105 expressed in pHM1 under the native promoter of avrRxo1; Sp^r^, Sm^r^	This work
pHMavrRxo1_(186-421)_	The fragment from 186 to 421 amino acids of AvrRxo1 from RS105 expressed in pHM1 under the native promoter of avrRxo1; Sp^r^, Sm^r^	This work
pHMavrRxo1_(K166N)_	pHM1 expressing AvrRxo1 from RS105 with K166N under the native promoter of avrRxo1; Sp^r^, Sm^r^	This work
pHMavrRxo1_(nls)_	pHM1 expressing AvrRxo1 from RS105 with R124A, R125A, K126A under the native promoter of avrRxo1; Sp^r^, Sm^r^	This work
pHMavrRxo1_(G165A)_	pHM1 expressing AvrRxo1 from RS105 with G165A under the native promoter of avrRxo1; Sp^r^, Sm^r^	This work
pHMavrRxo1_(H71A)_	pHM1 expressing AvrRxo1 from RS105 with H71A under the native promoter of avrRxo1; Sp^r^, Sm^r^	This work
pHMavrRxo1_(S344A)_	pHM1 expressing AvrRxo1 from RS105 with S344A under the native promoter of avrRxo1; Sp^r^, Sm^r^	This work
pGR106:avrRxo1	pGR106 expressing AvrRxo1 from different Xoc strains or different truncted fragments of *avrRxo1* under the CP promoter; Km^r^	This work
pGR106:avrRxo1_(1-159)_	The first 159 amino acids of AvrRxo1 from RS105 expressed in pGR106 under the CP promoter; Km^r^	This work
pGR106:avrRxo1_(1-193)_	The first 193 amino acids of AvrRxo1 from RS105 expressed in pGR106 under the CP promoter; Km^r^	This work
pGR106:avrRxo1_(1-278)_	The first 278 amino acids of AvrRxo1 from RS105 expressed in pGR106 under the CP promoter; Km^r^	This work
pGR106:avrRxo1_(1-373)_	The first 373 amino acids of AvrRxo1 from RS105 expressed in pGR106 under the CP promoter; Km^r^	This work
pGR106:avrRxo1_(1-412)_	The first 412 amino acids of AvrRxo1 from RS105 expressed in pGR106 under the CP promoter; Km^r^	This work
pGR106:avrRxo1_(1-417)_	The first 417 amino acids of AvrRxo1 from RS105 expressed in pGR106 under the CP promoter; Km^r^	This work
pGR106:avrRxo1_(1-418)_	The first 418 amino acids of AvrRxo1 from RS105 expressed in pGR106 under the CP promoter; Km^r^	This work
pGR106:avrRxo1_(1-419)_	The first 419 amino acids of AvrRxo1 from RS105 expressed in pGR106 under the CP promoter; Km^r^	This work
pGR106:avrRxo1_(1-420)_	The first 420 amino acids of AvrRxo1 from RS105 expressed in pGR106 under the CP promoter; Km^r^	This work
pGR106:avrRxo1_(17-421)_	The fragment from 17 to 421 amino acids of AvrRxo1 from RS105 expressed in pGR106 under the CP promoter; Km^r^	This work
pGR106:avrRxo1_(52-421)_	The fragment from 52 to 421 amino acids of AvrRxo1 from RS105 expressed in pGR106 under the CP promoter; Km^r^	This work
pGR106:avrRxo1_(109-421)_	The fragment from 109 to 421 amino acids of AvrRxo1 from RS105 expressed in pGR106 under the CP promoter; Km^r^	This work
pGR106:avrRxo1_(140-421)_	The fragment from 140 to 421 amino acids of AvrRxo1 from RS105 expressed in pGR106 under the CP promoter; Km^r^	This work
pGR106:avrRxo1_(186-421)_	The fragment from 186 to 421 amino acids of AvrRxo1 from RS105 expressed in pGR106 under the CP promoter; Km^r^	This work
pPIC3.5K:avrRxo1_RS105_	pPIC3.5K expressing AvrRxo1 from RS105 under the *AOX1* promoter; Km^r^	This work
pGR106:avrRxo1_(K166N)_	pGR106 expressing AvrRxo1 from RS105 with K166N under the CP promoter; Km^r^	This work
pGR106:avrRxo1_(nls)_	pGR106 expressing AvrRxo1 from RS105 with R124A, R125A, K126A under the CP promoter; Km^r^	This work
pGR106:avrRxo1_(G165A)_	pGR106 expressing AvrRxo1 from RS105 with G165A under the CP promoter; Km^r^	This work
pGR106:avrRxo1_(H71A)_	pGR106 expressing AvrRxo1 from RS105 with H71A under the CP promoter; Km^r^	This work
pGR106:avrRxo1_(S344A)_	pGR106 expressing AvrRxo1 from RS105 with S344A under the CP promoter; Km^r^	This work
pCX-DR	CaMV 35S promoter; ccdB; RFP; Km^r^	Chen *et al*., 2009
pCX-DR:avrRxo1	AvrRxo1 from RS105 fusion with RFP under the CaMV 35S promoter; Km^r^	This work
pCX-DR:avrRxo1_(52-421)_	The fragment from 52 to 421 amino acids of AvrRxo1 from RS105 fusion with RFP under the CaMV 35S promoter; Km^r^	This work
pCX-DR:avrRxo1_(109-421)_	The fragment from 109 to 421 amino acids of AvrRxo1 from RS105 fusion with RFP under the CaMV 35S promoter; Km^r^	This work
pGR106:avrRxo1_(S344A)_	pGR106 expressing AvrRxo1 from RS105 with S344A under the CP promoter; Km^r^	This work

Amp^r^, ampicillin resistance; CP, coat protein; Km^r^, kanamycin resistance; RFP, red fluorescent protein; Rif^r^, rifampicin resistance; Sm^r^, streptomycin; Sp^r^, spectinomycin.

Plasmid pGEM-avrRxo1 was constructed by T-A cloning in which the *avrRxo1* ORF1 was inserted into pGEM-T Easy vector (Promega, USA). The primers used to amplify the *avrRxo1* ORF1 were avrRxo1F1 and avrRxo1R1. The plasmid pHMavrRxo1 was constructed by introduction of a *Eco*RI and *Hin*dIII sequence containing the promoter and ORF1 of *avrRxo1* into the plasmid pHM1. The primers used to amplify the promoter and ORF1 of *avrRxo1* were PG-F and PG-R. The plasmid pGR106:avrRxo1 was constructed by introduction of a *Cla*I and *Not*I site sequence containing the ORF1 of *avrRxo1* into the plasmid pGR106. The primers used to amplify the ORF1 of *avrRxo1* were avrRxo1F2 and avrRxo1R2. The plasmid pPIC3.5K:avrRxo1 was constructed by introduction of a *Bam*HI site sequence containing the ORF1 of *avrRxo1* into the plasmid pPIC3.5K (Life technologies, USA). The primers used to amplify the ORF1 of *avrRxo1* were avrRxo1–3.5KF1 and avrRxo1–3.5KR1. The site-directed mutants of *avrRxo1* were made by overlapping PCR and ligated into the vectors pHM1 and pGR106 to produce the plasmids listed in Table 1. The truncated *avrRxo1* fragments were achieved by PCR using the primers listed in Table S1. All plasmids were validated by sequencing.

### Yeast strain and transformations

Yeast strain GS115 was cultured at 30°C in YPD medium (1% yeast extract, 2% peptone, 2% glucose) or in selective synthetic complete media lacking histidine (–his) to maintain the plasmid and was supplemented with 2% glucose or 1% methanol as carbon sources. LiAc/SS carrier DNA/PEG method was used to transfer plasmid DNA into yeast strain GS115 and was performed as previously described [49]. Plasmid DNA was linearised with *Sac*I for insertion at AOX1 to generate His^+^ Mut^+^ in GS115.

### Genomic DNA library construction

A genomic library of *X. oryzae* pv. *oryzicola* strain RS105, which contains one or more effectors suppressing the resistance in rice, was constructed in the cosmid vector pHM1 [11]. The genomic DNA of RS105 was purified with BacteriaGen DNA kit (CWBIO, China). Then, 1 µg of DNA was partially digested with *Sau*3AI (NEB, USA). The vector pHM1 was digested to completion with *Bam*HI (NEB, USA) and treated with calf intestinal alkaline phosphatase (NEB, USA). The genomic and vector DNA fragments were ligated with T4 DNA ligase (NEB, London) and transformed into DH5α. White colonies were selected on LB agar plates containing X-gal, IPTG and spectinomycin. For the library, more than 1000 clones were picked out with toothpicks and stored in three 384-well plates at −80°C. The size of the cloned genomic fragments ranged from 12 to 20 kb, and the average length was 17 kb, with approximately 3 times the coverage of the whole RS105 genome. The plasmids of each clone was purified and introduced into PXO99^A^ one by one.

### Bacterial transformations

Electrotransformation was used to transfer plasmid DNA into PXO99^A^ and performed as previously described [51]–[52]. Plasmid DNA was introduced into *E. coli* DH5α chemical competent cells (CWBIO, China). Plasmid DNA was introduced into *A. tumefaciens* strain GV3101 by electroporation as previously described [45].

### Plants culture and inoculation methods

The maize line B73 was used to test the expression of the *avrRxo*1 gene and the function of truncation and mutants of *avrRxo1* in Xoo strain PXO99^A^. Line B73 plants were grown in soil for 4 weeks, and the second and third leaves were inoculated with 48-h-old bacteria suspensions at 1×10^8^ CFU/mL. *N. benthamiana* were grown in a greenhouse at 25°C, with 70% relative humidity and 16 h photoperiod. Bacterial suspensions were infiltrated into the intercellular spaces with a needleless syringe. Plants were evaluated for the induction of HR 2 days post-infiltration.

### Transient expression of AvrRxo1 in *N. benthimiana*

*Agrobacterium* GV3101 containing *avrRxo1*, mutants, and vector pGR106 alone were resuspended with Agro-infiltration buffer (10mM MgCl_2_, 10 mM MES, pH 5.6, 200 µM Acetosyringone) and OD_600_ were adjusted to 0.5 (approximately 1 × 10^8^ CFU/ml). Bacterial suspensions were infiltrated into the 4-6 weeks old *N. benthamiana* leaves with a needleless syringe. The toxicity of the expressed protein was examined 5 days after infiltration.

### Subcellular localization of AvrRxo1 *in planta*

RFP was used as a reporter to investigate the subcellular localization of AvrRxo1 in planta. The fragments of AvrRxo1 full length, AvrRxo1(52–421), and AvrRxo1(109–421) were cloned in frame with red fluorescent protein in vector pCX-DR [51], generating pCX-DR:AvrRxo1(1–421), pCX-DR:AvrRxo1(52–421), and pCX-DR:AvrRxo1(109–421) (Table 1). The constructs described above were individually introduced into *A. tumefaciens* strain GV3101 and transient expression in *N. benthamiana* leaves. Two days after infiltration the treated leaves were observed using confocal laser scanning microscopy (Leica Microsystems, Solms, Germany).

## Supporting Information

Figure S1 **The normal expression of** ***avrRxo1*** **from different Xoc strains in infected** ***N. benthamiana*** **leaves were confirmed by RT-PCR.** Housekeeping gene EF1a was selected to normalize the samples Intrinsic disorder and globularity prediction of AvrRxo1 using GlobPlot software (http://globplot.embl.de/).
(TIF)

Figure S2 **Intrinsic disorder and globularity prediction of AvrRxo1 using GlobPlot software (** http://globplot.embl.de/ **).**
(TIF)

Figure S3 **Secondary structure analysis of AvrRxo1 using Jpred 3 software (** http://www.compbio.dundee.ac.uk/www-jpred/ **).** H indicates the α-helix; E indicates β-strand.
(TIF)

Table S1 **List of oligonucleotide primers used in this study.**
(DOC)

## References

[1] Ali GS, Reddy ASN (2008) PAMP-triggered immunity. Plant signaling & behavior 3:423–426. 19704848 10.4161/psb.3.6.5472 PMC2634595

[2] Galán JE, Collmer A (1999) Type III secretion machines: bacterial devices for protein delivery into host cells. Science 284:1322–1328. 10334981 10.1126/science.284.5418.1322

[3] Jones JD, Dangl JL (2006) The plant immune system. Nature 444:323–329. 17108957 10.1038/nature05286

[4] annual review

[5] Zhu W, MaGbanua MM, White FF (2000) Identification of Two Novelhrp-Associated Genes in the hrp Gene Cluster of Xanthomonas oryzae pv. oryzae. Journal of bacteriology 182:1844–1853. 10714988 10.1128/jb.182.7.1844-1853.2000 PMC101866

[6] Nino-Liu DO, Ronald PC, Bogdanove AJ (2006) Xanthomonas oryzae pathovars: model pathogens of a model crop. Mol Plant Pathol 7:303–324. 20507449 10.1111/j.1364-3703.2006.00344.x

[7] Zhang H, Wang S (2013) Rice versus Xanthomonas oryzae pv. oryzae: a unique pathosystem. Curr Opin Plant Biol 16:188–195. 23466254 10.1016/j.pbi.2013.02.008

[8] Sugio A, Yang B, Zhu T, White FF (2007) Two type III effector genes of Xanthomonas oryzae pv. oryzae control the induction of the host genes OsTFIIAgamma1 and OsTFX1 during bacterial blight of rice. Proc Natl Acad Sci U S A 104:10720–10725. 17563377 10.1073/pnas.0701742104 PMC1965579

[9] Chen LQ, Hou BH, Lalonde S, Takanaga H, Hartung ML, et al (2010) Sugar transporters for intercellular exchange and nutrition of pathogens. Nature 468:527–532. 21107422 10.1038/nature09606 PMC3000469

[10] Chu Z, Yuan M, Yao J, Ge X, Yuan B, et al (2006) Promoter mutations of an essential gene for pollen development result in disease resistance in rice. Genes Dev 20:1250–1255. 16648463 10.1101/gad.1416306 PMC1472899

[11] Yang B, Sugio A, White FF (2006) Os8N3 is a host disease-susceptibility gene for bacterial blight of rice. Proc Natl Acad Sci U S A 103:10503–10508. 16798873 10.1073/pnas.0604088103 PMC1502487

[12] Yuan M, Chu Z, Li X, Xu C, Wang S (2010) The bacterial pathogen Xanthomonas oryzae overcomes rice defenses by regulating host copper redistribution. Plant Cell 22:3164–3176. 20852017 10.1105/tpc.110.078022 PMC2965554

[13] Antony G, Zhou J, Huang S, Li T, Liu B, et al (2010) Rice xa13 recessive resistance to bacterial blight is defeated by induction of the disease susceptibility gene Os-11N3. Plant Cell 22:3864–3876. 21098734 10.1105/tpc.110.078964 PMC3015117

[14] Yu Y, Streubel J, Balzergue S, Champion A, Boch J, et al (2011) Colonization of rice leaf blades by an African strain of Xanthomonas oryzae pv. oryzae depends on a new TAL effector that induces the rice nodulin-3 Os11N3 gene. Mol Plant Microbe Interact 24:1102–1113. 21679014 10.1094/MPMI-11-10-0254

[15] White FF, Potnis N, Jones JB, Koebnik R (2009) The type III effectors of Xanthomonas. Mol Plant Pathol 10:749–766. 19849782 10.1111/j.1364-3703.2009.00590.x PMC6640274

[16] Song C, Yang B (2010) Mutagenesis of 18 type III effectors reveals virulence function of XopZ(PXO99) in Xanthomonas oryzae pv. oryzae. Mol Plant Microbe Interact 23:893–902. 20521952 10.1094/MPMI-23-7-0893

[17] Akimoto-Tomiyama C, Furutani A, Tsuge S, Washington EJ, Nishizawa Y, et al (2012) XopR, a type III effector secreted by Xanthomonas oryzae pv. oryzae, suppresses microbe-associated molecular pattern-triggered immunity in Arabidopsis thaliana. Mol Plant Microbe Interact 25:505–514. 22204644 10.1094/MPMI-06-11-0167

[18] Zhao S, Mo WL, Wu F, Tang W, Tang JL, et al (2013) Identification of non-TAL effectors in Xanthomonas oryzae pv. oryzae Chinese strain 13751 and analysis of their role in the bacterial virulence. World J Microbiol Biotechnol 29:733–744. 23296915 10.1007/s11274-012-1229-5

[19] Sinha D, Gupta MK, Patel HK, Ranjan A, Sonti RV (2013) Cell Wall Degrading Enzyme Induced Rice Innate Immune Responses Are Suppressed by the Type 3 Secretion System Effectors XopN, XopQ, XopX and XopZ of Xanthomonas oryzae pv. oryzae. PLoS One 8:e75867. 24086651 10.1371/journal.pone.0075867 PMC3784402

[20] Kang MJ, Shim JK, Cho MS, Seol YJ, Hahn JH, et al (2008) Specific detection of Xanthomonas oryzae pv. oryzicola in infected rice plant by use of PCR assay targeting a membrane fusion protein gene. J Microbiol Biotechnol 18:1492–1495. 18852502

[21] Guo X, Zou H, Li Y, Zou L, Chen G (2010) [HrpD6 gene determines Xanthomonas oryzae pv. oryzae to trigger hypersensitive response in tobacco and pathogenicity in rice]. Wei Sheng Wu Xue Bao 50:1155–1163. 21090255

[22] Zhao B, Ardales EY, Raymundo A, Bai J, Trick HN, et al (2004) The avrRxo1 gene from the rice pathogen Xanthomonas oryzae pv. oryzicola confers a nonhost defense reaction on maize with resistance gene Rxo1. Mol Plant Microbe Interact 17:771–779. 15242171 10.1094/MPMI.2004.17.7.771

[23] Zhao B, Lin X, Poland J, Trick H, Leach J, et al (2005) A maize resistance gene functions against bacterial streak disease in rice. Proc Natl Acad Sci U S A 102:15383–15388. 16230639 10.1073/pnas.0503023102 PMC1266081

[24] Salomon D, Dar D, Sreeramulu S, Sessa G (2011) Expression of Xanthomonas campestris pv. vesicatoria type III effectors in yeast affects cell growth and viability. Mol Plant Microbe Interact 24:305–314. 21062109 10.1094/MPMI-09-10-0196

[25] Cui Y, Zou L, Zou H, Li Y, Zakria M, et al (2013) HrpE3 is a type III effector protein required for full virulence of Xanthomonas oryzae pv. oryzicola in rice. Mol Plant Pathol 14:678–692. 23672717 10.1111/mpp.12039 PMC6638819

[26] Makino S, Sugio A, White F, Bogdanove AJ (2006) Inhibition of resistance gene-mediated defense in rice by Xanthomonas oryzae pv. oryzicola. Mol Plant Microbe Interact 19:240–249. 16570654 10.1094/MPMI-19-0240

[27] Cole C, Barber JD, Barton GJ (2008) The Jpred 3 secondary structure prediction server. Nucleic Acids Res 36:W197–201. 18463136 10.1093/nar/gkn238 PMC2447793

[28] Linding R, Russell RB, Neduva V, Gibson TJ (2003) GlobPlot: Exploring protein sequences for globularity and disorder. Nucleic Acids Res 31:3701–3708. 12824398 10.1093/nar/gkg519 PMC169197

[29] Bogdanove AJ, Koebnik R, Lu H, Furutani A, Angiuoli SV, et al (2011) Two new complete genome sequences offer insight into host and tissue specificity of plant pathogenic Xanthomonas spp. J Bacteriol 193:5450–5464. 21784931 10.1128/JB.05262-11 PMC3187462

[30] Bartetzko V, Sonnewald S, Vogel F, Hartner K, Stadler R, et al (2009) The Xanthomonas campestris pv. vesicatoria type III effector protein XopJ inhibits protein secretion: evidence for interference with cell wall-associated defense responses. Mol Plant Microbe Interact 22:655–664. 19445590 10.1094/MPMI-22-6-0655

[31] Canonne J, Marino D, Jauneau A, Pouzet C, Briere C, et al (2011) The Xanthomonas type III effector XopD targets the Arabidopsis transcription factor MYB30 to suppress plant defense. Plant Cell 23:3498–3511. 21917550 10.1105/tpc.111.088815 PMC3203416

[32] Kim JG, Li X, Roden JA, Taylor KW, Aakre CD, et al (2009) Xanthomonas T3S Effector XopN Suppresses PAMP-Triggered Immunity and Interacts with a Tomato Atypical Receptor-Like Kinase and TFT1. Plant Cell 21:1305–1323. 19366901 10.1105/tpc.108.063123 PMC2685636

[33] Metz M, Dahlbeck D, Morales CQ, Al Sady B, Clark ET, et al (2005) The conserved Xanthomonas campestris pv. vesicatoria effector protein XopX is a virulence factor and suppresses host defense in Nicotiana benthamiana. Plant J 41:801–814. 15743446 10.1111/j.1365-313X.2005.02338.x

[34] Buell CR, Joardar V, Lindeberg M, Selengut J, Paulsen IT, et al (2003) The complete genome sequence of the Arabidopsis and tomato pathogen Pseudomonas syringae pv. tomato DC3000. Proc Natl Acad Sci U S A 100:10181–10186. 12928499 10.1073/pnas.1731982100 PMC193536

[35] Alfano JR, Collmer A (2004) Type III secretion system effector proteins: double agents in bacterial disease and plant defense. Annu Rev Phytopathol 42:385–414. 15283671 10.1146/annurev.phyto.42.040103.110731

[36] Abramovitch RB, Kim YJ, Chen S, Dickman MB, Martin GB (2003) Pseudomonas type III effector AvrPtoB induces plant disease susceptibility by inhibition of host programmed cell death. EMBO J 22:60–69. 12505984 10.1093/emboj/cdg006 PMC140047

[37] Espinosa A, Guo M, Tam VC, Fu ZQ, Alfano JR (2003) The Pseudomonas syringae type III-secreted protein HopPtoD2 possesses protein tyrosine phosphatase activity and suppresses programmed cell death in plants. Mol Microbiol 49:377–387. 12828636 10.1046/j.1365-2958.2003.03588.x

[38] Kang L, Tang X, Mysore KS (2004) Pseudomonas Type III effector AvrPto suppresses the programmed cell death induced by two nonhost pathogens in Nicotiana benthamiana and tomato. Mol Plant Microbe Interact 17:1328–1336. 15597738 10.1094/MPMI.2004.17.12.1328

[39] Lopez-Solanilla E, Bronstein PA, Schneider AR, Collmer A (2004) HopPtoN is a Pseudomonas syringae Hrp (type III secretion system) cysteine protease effector that suppresses pathogen-induced necrosis associated with both compatible and incompatible plant interactions. Mol Microbiol 54:353–365. 15469508 10.1111/j.1365-2958.2004.04285.x

[40] Zhang J, Shao F, Li Y, Cui H, Chen L, et al (2007) A Pseudomonas syringae effector inactivates MAPKs to suppress PAMP-induced immunity in plants. Cell Host Microbe 1:175–185. 18005697 10.1016/j.chom.2007.03.006

[41] Gohre V, Spallek T, Haweker H, Mersmann S, Mentzel T, et al (2008) Plant pattern-recognition receptor FLS2 is directed for degradation by the bacterial ubiquitin ligase AvrPtoB. Curr Biol 18:1824–1832. 19062288 10.1016/j.cub.2008.10.063

[42] Xiang T, Zong N, Zou Y, Wu Y, Zhang J, et al (2008) Pseudomonas syringae effector AvrPto blocks innate immunity by targeting receptor kinases. Curr Biol 18:74–80. 18158241 10.1016/j.cub.2007.12.020

[43] Li W, Xu YP, Zhang ZX, Cao WY, LiF, Zhou X, et al (2012) Identification of genes required for nonhost resistance to Xanthomonas oryzae pv. oryzae reveals novel signaling components. PloS one 7:e42796. 22912739 10.1371/journal.pone.0042796 PMC3418293

[44] Bahadur RP, Basak J (2014) Molecular modeling of protein-protein interaction to decipher the structural mechanism of nonhost resistance in rice. J Biomol Struct Dyn 32:669–681. 23659345 10.1080/07391102.2013.787370

[45] Shan L, Thara VK, Martin GB, Zhou JM, Tang X (2000) The pseudomonas AvrPto protein is differentially recognized by tomato and tobacco and is localized to the plant plasma membrane. Plant Cell 12:2323–2338. 11148281 10.1105/tpc.12.12.2323 PMC102221

[46] Maurer-Stroh S, Eisenhaber F (2004) Myristoylation of viral and bacterial proteins. Trends in microbiology 12:178–185. 15051068 10.1016/j.tim.2004.02.006

[47] Szurek B, Marois E, Bonas U, Van den Ackerveken G (2001) Eukaryotic features of the Xanthomonas type III effector AvrBs3: protein domains involved in transcriptional activation and the interaction with nuclear import receptors from pepper. Plant J 26:523–534. 11439138 10.1046/j.0960-7412.2001.01046.x

[48] Van den Ackerveken G, Marois E, Bonas U (1996) Recognition of the bacterial avirulence protein AvrBs3 occurs inside the host plant cell. Cell 87:1307–1316. 8980236 10.1016/s0092-8674(00)81825-5

[49] Gietz RD, Schiestl RH (2007) Frozen competent yeast cells that can be transformed with high efficiency using the LiAc/SS carrier DNA/PEG method. Nat Protoc 2:1–4. 17401330 10.1038/nprot.2007.17

[50] Choi SH, Leach JE (1994) Identification of the XorII methyltransferase gene and a vsr homolog from Xanthomonas oryzae pv. oryzae. Mol Gen Genet 244:383–390. 8078464 10.1007/BF00286690

[51] Chen S, Songkumarn P, Liu J, Wang GL (2009) A versatile zero background T-vector system for gene cloning and functional genomics. Plant Physiol 150:1111–1121. 19403729 10.1104/pp.109.137125 PMC2705043

[52] Feng W, Chang Q, Yang L, Ding X, Chu Z (2013) Screening and detection of diagnostic molecular markers to distinguish Xanthomonas oryzea pv. oryzae from Xanthomonas oryzea pv. oryzicola. Acta Phytopathologica Sinica 43:581–589.

