# Supplementary figures and images for: Domain Dissection of AvrRxo1 for Suppressor, Avirulence and Cytotoxicity Functions
Source: PLoS One. 2014 Dec 1;9(12):e113875. doi: 10.1371/journal.pone.0113875 (PMC4250038; doi:10.1371/journal.pone.0113875)

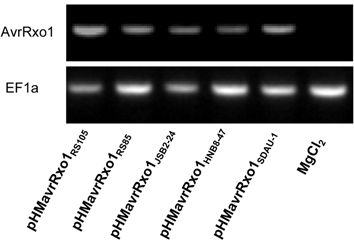

Supplement: Figure S1 — The normal expression of avrRxo1 from different Xoc strains in infected N. benthamiana leaves were confirmed by RT-PCR. Housekeeping gene EF1a was selected to normalize the samples Intrinsic disorder and globularity prediction of AvrRxo1 using GlobPlot software (http://globplot.embl.de/). (TIF) [file pone.0113875.s001.tif]

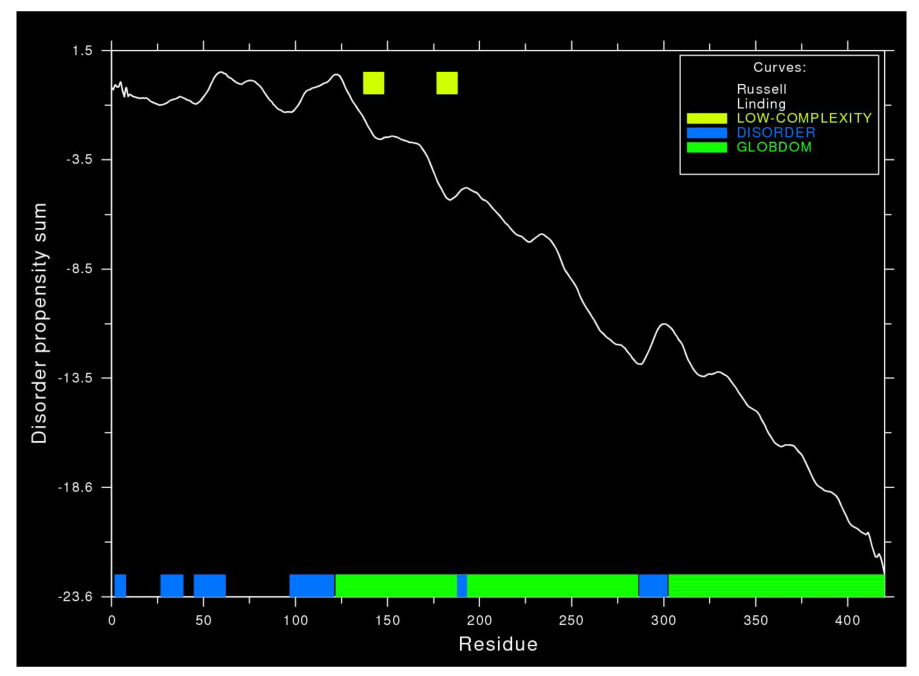

Supplement: Figure S2 — Intrinsic disorder and globularity prediction of AvrRxo1 using GlobPlot software ( http://globplot.embl.de/ ). (TIF) [file pone.0113875.s002.tif]

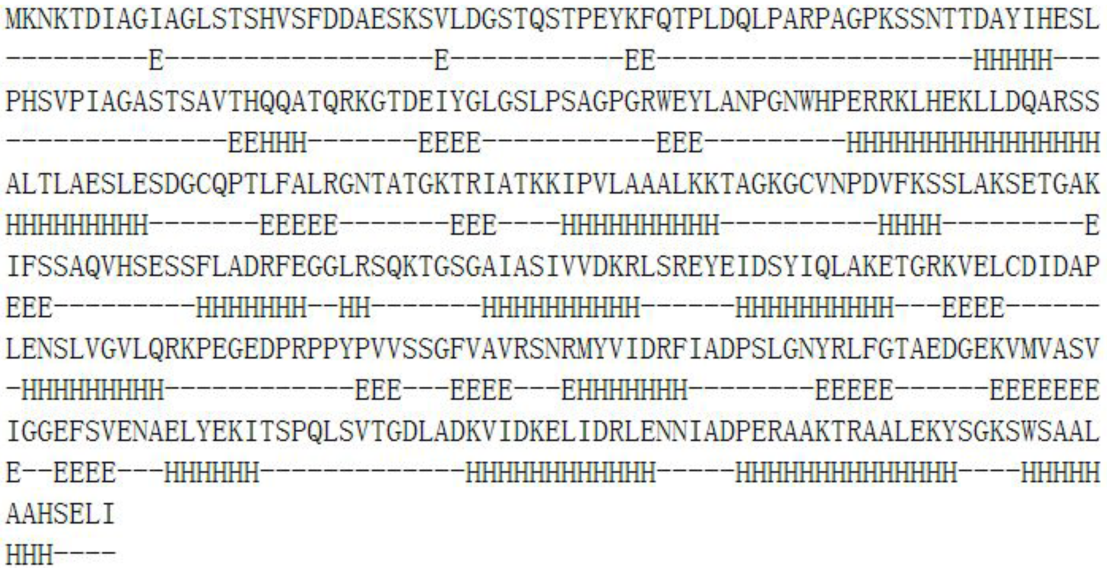

Supplement: Figure S3 — Secondary structure analysis of AvrRxo1 using Jpred 3 software ( http://www.compbio.dundee.ac.uk/www-jpred/ ). H indicates the α-helix; E indicates β-strand. (TIF) [file pone.0113875.s003.tif]
