# Supplementary material for: Domain Dissection of AvrRxo1 for Suppressor, Avirulence and Cytotoxicity Functions
Source: PLoS One. 2014 Dec 1;9(12):e113875. doi: 10.1371/journal.pone.0113875 (PMC4250038; doi:10.1371/journal.pone.0113875)
Supplement: Table S1 — List of oligonucleotide primers used in this study. (DOC) [file pone.0113875.s004.doc]

Table S1. List of oligonucleotide primers used in this study

| **Primers** | **Sequence shown in 5’→3’ orientation** | **Description** |
| --- | --- | --- |
| avrRxo1F1 | CTTCGTTCGCTGCTCATA | Amplifies a 1400 bp fragment containing *avrRxo1* in different Xoc strains |
| avrRxo1R1 | ATTGCTTTTCCCCTTTCA |
| PG-F | ATGGAATTCTCGCTGGGCTGCTCTACCTC | Amplifies promoter and ORF1 of *avrRxo1*; *Eco*RI and *Hind*III sites are underscored |
| PG-R | ATGAAGCTTGCGTCTGCGCCTGTCAAAGT |
| avrRXO1F2 | ATGATCGATCTTCGTTCGCTGCTCATA | Amplifies ORF1 of *avrRxo1*; *Cla*I and *Not*I sites are underscored |
| avrRXO1R2 | ATTGCGGCCGCATTGCTTTTCCCCTTTCA |
| avrRXO1-3.5KF1 | TGGGGATCCATGAAAAACAAGACAGACATT | Amplifies ORF1 of *avrRxo1*; *Bam*HI sites are underscored |
| avrRXO1-3.5KR1 | ATGGGATCCTCAAATTAGCTCGCTGTGAG |
| avrRxo1-cR1 | ATGAAGCTTTTAGCGCAGCGCAAACAATG | 3’ primer deletion C terminus of *avrRxo1* at 159 amino acid; *Hind*III site is underscored |
| avrRxo1-cR2 | ATGAAGCTTTTAATCTGGATTCACGCAACCTTTC | 3’ primer deletion C terminus of *avrRxo1* at 193 amino acid; *Hind*III site is underscored |
| avrRxo1-cR3 | ATGAAGCTTTTAAGCCAAATCGCCCGTAACC | 3’ primer deletion C terminus of *avrRxo1* at 278 amino acid; *Hind*III site is underscored |
| avrRxo1-cR4 | ATGAAGCTTTTACTCTAGGGGCGCGTCGATAT | 3’ primer deletion C terminus of *avrRxo1* at 373 amino acid; *Hind*III site is underscored |
| avrRxo1-cR5 | ATGAAGCTTTCAGGCCGACCATGACTTACCG | 3’ primer deletion C terminus of *avrRxo1* at 412 amino acid; *Hind*III site is underscored |
| avrRxo1-cR6 | ATGAAGCTTTCAGTGAGCAGCTAGCGCGGC | 3’ primer deletion C terminus of *avrRxo1* at 417 amino acid; *Hind*III site is underscored |
| avrRxo1-cR7 | ATGAAGCTTTCACTCGCTGTGAGCAGCTAGCGC | 3’ primer deletion C terminus of *avrRxo1* at 419 amino acid; HindIII site is underscored |
| avrRxo1-cR8 | ATGAAGCTTTCATAGCTCGCTGTGAGCAGCTAGC | 3’ primer deletion C terminus of *avrRxo1* at 420 amino acid; *Hind*III site is underscored |
| avrRxo1-cR9 | ATTGCGGCCGCTTAGCGCAGCGCAAACAATG | 3’ primer deletion C terminus of *avrRxo1* at 159 amino acid; *Not*I site is underscored |
| avrRxo1-cR10 | ATTGCGGCCGCTTAATCTGGATTCACGCAACCTTTC | 3’ primer deletion C terminus of *avrRxo1* at 193 amino acid; *Not*I site is underscored |
| avrRxo1-cR11 | ATTGCGGCCGCTTAAGCCAAATCGCCCGTAACC | 3’ primer deletion C terminus of *avrRxo1* at 278 amino acid; *Not*I site is underscored |
| avrRxo1-cR12 | ATTGCGGCCGCTTACTCTAGGGGCGCGTCGATAT | 3’ primer deletion C terminus of *avrRxo1* at 373 amino acid; *Not*I site is underscored |
| avrRxo1-cR13 | ATTGCGGCCGCTCAGGCCGACCATGACTTACCG | 3’ primer deletion C terminus of *avrRxo1* at 412 amino acid; *Not*I site is underscored |
| avrRxo1-cR14 | ATTGCGGCCGCTCAGTGAGCAGCTAGCGCGGC | 3’ primer deletion C terminus of *avrRxo1* at 417 amino acid; *Not*I site is underscored |
| avrRxo1-cR15 | ATTGCGGCCGCTCACTCGCTGTGAGCAGCTAGCGC | 3’ primer deletion C terminus of *avrRxo1* at 419 amino acid; *Not*I site is underscored |
| avrRxo1-cR16 | ATTGCGGCCGCTCATAGCTCGCTGTGAGCAGCTAGC | 3’ primer deletion C terminus of *avrRxo1* at 420 amino acid; *Not*I site is underscored |
| avrRxo1-nF1 | ATGATCGATATGGTTTCCTTTGATGACGCAGA | 5’ primer deletion N terminus of *avrRxo1* at 17 amino acid; *Cla*I site is underscored |
| avrRxo1-nF2 | ATGATCGATATGGCTGGTCCGAAGAGCAGTAAC | 5’ primer deletion N terminus of *avrRxo1* at 52 amino acid; *Cla*I site is underscored |
| avrRxo1-nF3 | ATGATCGATATGCGATGGGAGTATCTTGCGAAT | 5’ primer deletion N terminus of *avrRxo1* at 109 amino acid; *Cla*I site is underscored |
| avrRxo1-nF4 | ATGATCGATATGACCCTGGCAGAATCCCTT | 5’ primer deletion N terminus of *avrRxo1* at 140 amino acid; *Cla*I site is underscored |
| avrRxo1-nF5 | ATGATCGATATGAAAGGTTGCGTGAATCCAG | 5’ primer deletion N terminus of *avrRxo1* at 186 amino acid; *Cla*I site is underscored |
| avrRxo1-nF6 | ATGGGTACCATGGGCATAGCCGGACTTTCT | 5’ primer deletion N terminus of *avrRxo1* at 8 amino acid; *Kpn*I site is underscored |
| avrRxo1-nF7 | ATGGGTACCATGGTTTCCTTTGATGACGCAGA | 5’ primer deletion N terminus of *avrRxo1* at 17 amino acid; *Kpn*I site is underscored |
| avrRxo1-nF8 | ATGGGTACCATGGCTGGTCCGAAGAGCAGTAAC | 5’ primer deletion N terminus of *avrRxo1* at 52 amino acid; *Kpn*I site is underscored |
| avrRxo1-nF9 | ATGGGTACCATGCGATGGGAGTATCTTGCGAAT | 5’ primer deletion N terminus of *avrRxo1* at 109 amino acid; *Kpn*I site is underscored |
| avrRxo1-nF10 | ATGGGTACCATGACCCTGGCAGAATCCCTT | 5’ primer deletion N terminus of *avrRxo1* at 140 amino acid; *Kpn*I site is underscored |
| avrRxo1-nF11 | ATGGGTACCATGAAAGGTTGCGTGAATCCAG | 5’ primer deletion N terminus of *avrRxo1* at 186 amino acid; *Kpn*I site is underscored |
| G165A-F1 | CGCAACGGCTAAAACACGG | overlapping primers to get mutation G165A |
| G165A-R1 | CCGTGTTTTAGCCGTTGCG |
| K166N-F1 | CGCAACGGGTAATACACGGATT | overlapping primers to get mutation K166N |
| K166N-R1 | AATCCGTGTATTACCCGTTGCG |
| H71A-F1 | TCACTGCCGGCTTCGGTTCC | overlapping primers to get mutation H71A |
| H71A-R1 | GGAACCGAAGCCGGCAGTGA |
| NLS-MF | AGGGCGGTGCGTTACACGAA | overlapping primers to get mutation R124A, R125A, K126A |
| NLS-MR | TAACGCACCGCCCTCGGGAT |
